# Users’ Modifications to Electronic Nicotine Delivery Systems (ENDS): Interviews with ENDS Enthusiasts

**DOI:** 10.3390/ijerph17030918

**Published:** 2020-02-02

**Authors:** Yachao Li, Robert T. Fairman, Victoria Churchill, David L. Ashley, Lucy Popova

**Affiliations:** 1 Department of Communication Studies and Department of Public Health, The College of New Jersey, Ewing, NJ 08628, USA; liya@tcnj.edu; 2 School of Public Health, Georgia State University, Atlanta, GA 30302, USA; rfairman1@student.gsu.edu (R.T.F.); vchurchill1@student.gsu.edu (V.C.); dashley4@gsu.edu (D.L.A.)

**Keywords:** batteries, coils, e-liquids, ENDS modifications, vaping

## Abstract

Users’ modifications to electronic nicotine delivery systems (ENDS) products could increase initiation, inhibit cessation, or change the toxicity of the product. This study aims to begin to identify consumers’ common ENDS modification behaviors. We conducted audio-recorded, in-depth one-on-one interviews with 13 adult ENDS users in the metropolitan Atlanta area, who self-reported extensive modification experience. Modifications to coils, batteries, and e-liquids were commonly mentioned. Participants indicated that users modified devices to produce large clouds, change levels of nicotine delivery, alter tastes of e-liquids, and experience different throat hits. Because manufacturers have changed product characteristics to be in line with consumer preferences, interviewees indicated that fewer users currently engage in modifications to coils and batteries compared to the more widespread practice a few years ago. Hobbyists continue to perform modifications and many users continue to misuse or abuse e-liquids, despite the view that fewer users currently alter their ENDS than in the past. The Food and Drug Administration (FDA) regulatory actions that limit certain product characteristics may unintentionally increase the likelihood that users will once again make more extensive modifications to their products, and this should be considered as part of the FDA’s regulatory decision-making process.

## 1. Introduction

The use of electronic nicotine delivery systems (ENDS), also called e-cigarettes, e-hookahs, and vape devices, is a growing public health issue in the United States [1]. Almost one in 20 U.S. adults (10.8 million) are using ENDS, among whom over half are under 35 years old [2]. As of January 7, 2020, a total of 2602 hospitalized cases of lung injury associated with ENDS use have been reported to the Centers for Disease Control and Prevention (CDC), and 57 deaths have been confirmed [3]. These cases have been linked to the use of vitamin E acetate in the tetrahydrocannabinol (THC) used in ENDS [4]. Growing evidence suggests that, while ENDS may be used by some smokers to quit smoking [5], ENDS use has both short-term and long-term health risks [6,7,8]. Moreover, ENDS products often include highly modifiable features, allowing users to alter device, liquid, and aerosol characteristics, which may lead to even more harmful effects [9].

ENDS modifications include product misuse and tampering unintended by the manufacturers, as well as alteration, customization, adjustment, and user choice of e-liquid or accessories made within manufacturer parameters. For instance, users may modify the liquid materials to be aerosolized, such as making their own e-juice or adding substances such as cannabis. Users may substitute liquids intended by the manufacturer with materials of unknown origin and composition. Users may also modify the voltage of the heating coil, resulting in higher nicotine delivery and exposure to higher levels of other harmful substances in the aerosol [10]. In addition, the ability to customize or modify flavors and nicotine levels is an attractive feature of ENDS [9]. The availability of certain flavors also increases the likelihood of youth use [1]. Thus, regardless of whether the modifications are options provided by the manufacturers as a choice for ENDS users (e.g., switching flavors or nicotine concentrations) or are not intended by manufacturers (e.g., making coils, changing batteries), ENDS modifications could change the toxicity of the product, increase initiation, and inhibit cessation of the product use.

However, very little is known about modifications made by users to ENDS. The Population Assessment of Tobacco and Health (PATH) survey has two items related to modifications: whether participants can change the voltage on their ENDS devices, and if they have ever modified their ENDS; in which, participants can only answer yes or no. This data does not have the degree of depth and specificity needed to adequately inform product standards or marketing decisions for specific product applications to minimize misuse of ENDS. Indeed, to the best of our knowledge, no studies have used these data from the PATH survey. Thus, the purpose of this study is to identify and understand consumers’ common ENDS modification behaviors. We conducted in-depth one-on-one interviews with 13 ENDS enthusiasts (i.e., people who are highly interested in ENDS, use the products frequently, and have modified them in the past) in the metropolitan Atlanta area to explore how and why users modify their ENDS devices and develop hypotheses for quantitative surveys.

## 2. Materials and Methods

### 2.1. Participants

John Snow Inc. (JSI), a public health consulting firm, utilized both online (e.g., Craigslist, Facebook advertisements) and offline (e.g., flyers in vaping shops, interviewee referrals) recruitment strategies to invite potential participants. Interested individuals completed an online screener that assessed their demographics, tobacco use, and experience with modifying ENDS products. Participants were considered eligible for this study if they (a) were 18 years or older, (b) self-reported currently using ENDS every day or some days, and (c) stated that they had experience with modifying the ENDS devices they routinely used. For the purpose of this study, people who met our eligibility criteria were called “ENDS enthusiasts”. A total of 112 individuals completed the screener, 43 of whom were eligible and were contacted for an interview. We interviewed 12 ENDS enthusiasts from the metropolitan Atlanta area. This sample size is typically sufficient to reach theme saturation [11]. A review of an additional interview (number 13), indeed, showed that no new themes emerged and thus, data saturation was reached. All 13 interviews were included in the data analysis. Each participant received $50 compensation.

### 2.2. Interviews

JSI conducted 13 semi-structured interviews between January and May 2019. An experienced moderator led each interview, which was audio-recorded. Research team members also observed the interviews and took notes. The interviews ranged in length between 15 and 62 min. Most sessions were conducted at various vape shops for participants’ convenience. Some interviews were conducted in a private meeting space near the JSI Atlanta office. All participants signed informed consent and the study was approved by the Institutional Review Board of Georgia State University. Participant names have been substituted with pseudonyms to maintain confidentiality. Semi-structured interviews explored different ways participants modified their ENDS, the reasons for these modifications, and personal stories and experiences related to modified ENDS.

### 2.3. Data Analysis

We utilized a thematic analysis [12] approach to analyze the data. The interviews were transcribed, yielding 239 pages of single-spaced data. The first author, Y.L., then listened to all the interviews and read the transcripts simultaneously to verify the accuracy of transcription and develop an overall picture of the participants’ responses. Next, each research team member read two to three interviews thoroughly and wrote research memos. The team met regularly to discuss emerging themes and develop an initial codebook. R.T.F. and V.C. then independently coded two interviews. Coding discrepancies were discussed and resolved in group meetings, and the codebook was finalized. R.T.F. then completed the coding of the rest of the data in NVivo 12. After all interviews were coded, the research team engaged in in-depth readings of the coded transcripts and wrote memos that discussed the themes. Y.L. then read all the memos and corresponding transcripts and synthesized the findings.

## 3. Results

Participants were 31% cisgender women and 69% cisgender men; 46% African American, 31% European American, and 23% other races; 46% had a bachelor’s or graduate degree; and 31%, 54%, and 15% were aged 18–24, 25–44, and 45–64 years, respectively. No participants self-identified as Hispanic or Latino. All 13 participants were daily ENDS users and only one participant (Daniel) was a past 30-day dual user of cigarettes and ENDS. All participants modified the ENDS devices they routinely used. Table 1 presents the pseudonym, demographic information, tobacco use, and ENDS modification practices of each participant. Information about vaping initiation was retrieved from the interviews.

### 3.1. Types of Users’ Modifications to ENDS

Overall, participants engaged in various ENDS modification practices. Modifications to coils and batteries were most commonly discussed. Users also refilled closed pods, changed e-liquids with different flavors, or even created their own e-liquids.

#### 3.1.1. Coils

Of the 13 participants, all but one mentioned that building and replacing coils were the most common and intensive modifications. Building coils refers to making users’ own coils from basic materials. While the exact techniques for coil building for each type of ENDS device differs, the major steps generally involve preparing a piece of wire, wrapping the wire around a small screwdriver a specific number of times, installing and testing the coils, and wicking the coils (inserting a wick). Some participants expressed that although building coils appears to be straightforward, it is actually quite time-consuming and requires intensive self-learning. Thus, instead of building their own coils, more often users replaced existing coils with pre-built coils from manufacturers. The new coils, either built or replacement, usually have different levels of wattage range, numbers of wire wraps, wire gauge, and/or wire materials (e.g., nickel versus stainless steel) than the old ones. Anthony, a former vape shop worker, said he and other retailers always recommended customers change coils to meet their individual needs. In addition, George indicated that some users built and sold coils to other consumers full-time.

Participants mentioned several reasons for modifying their coils. First, users built and replaced different coils so that they could experience various cloud densities. Several interviewees explained that coils with different forms of wire wrapping yield different heating speeds, which lead to more or less dense clouds. Anthony also stated that some ENDS users participated in cloud competitions at which people competed for the thickness and density of their clouds.

Moreover, users sometimes preferred different levels of nicotine delivery and thus, switched between different types of coils with various wattage ranges, such as smaller coils with higher resistance for salt nicotine and bigger coils with lower resistance for regular or freebase nicotine. Similarly, altering the number of wire wraps, wire gauge, and wire materials results in different wattage ranges of coils, which affects the flavor and tastes of e-liquids. Jacob said, “Coils were so bad previously. They were fine, but the flavor wasn’t there. You had limited (control) over the cloud production, how much power you could push through the coil without burning the coil. So most of the people that got into building their own devices got into there so they can do higher wattage, lower nicotine, bigger cloud”. In addition, a couple of participants modified coils to experience stronger or weaker throat hits, or different sensory perceptions. Anthony explained, “If you want a smoother hit, you want to go something closer to a mesh coil, which has mesh fibers on the inside so it’s better airflow and it’s a smoother hit, rather than the T12 or the T10, which has higher wattage coils, which is a stronger hit, higher wattage”. Finally, Josh commented that some hobbyists built their own coils for aesthetical reasons, called “coil art”.

#### 3.1.2. Cotton Wicks

More than half of the participants mentioned that they switched between different types of cotton wicks to identify the right choice. Three categories were discussed, including regular cotton, organic cotton, and Japanese cotton. The regular cotton refers to cotton materials purchased in regular stores that are not intended for vaping. Several participants expressed that regular cotton is often bleached and contains harmful chemicals, which makes the products unsafe and affects the tastes of e-liquids. Organic cotton is unbleached cotton designed for vaping. Charlotte pointed out a specific product named “cotton bacon” that is often in strips, offers faster e-liquid absorption, and has higher heat resistance. The last category is Japanese cotton, which is often pad-shaped, produced in Japan, and originally used for skincare purposes. Anthony believed that Japanese cotton has the finest quality and lasts longer than other cotton wicks. He also said, “If you had regular cotton where you’re buying from the store, it wasn’t organic, you would blow the cotton and that was to get the chemicals off of the cotton before you would put it in your mod”. Thus, users mainly modified their cotton wicks for safety, taste, and quality reasons.

#### 3.1.3. Batteries

All but one participant mentioned modifying batteries in their ENDS devices. Several practices were commonly stated, including replacing an old battery with a new battery of the same type, upgrading from internal to external batteries, switching smaller mods with bigger ones, and installing specific batteries into devices that were not designed for those types of batteries.

Batteries can become worn out and malfunction as they age. Thus, for safety concerns, many users replaced the worn batteries with new ones. Alex said, “There have been rumors that vapes are expanding and exploding because of a battery malfunction, so if your battery is nice and clean like this, then it’s safe to go… If it’s torn, no matter if it’s torn by the littlest bit, I will just get a whole new battery itself”. In addition, users sometimes upgraded their old batteries to the same type of products with better quality. Jack explained that as lower-end batteries are running low, the throat hits become weaker. Therefore, some users upgraded to higher-end batteries that last longer and keep the throat hits consistent even when the batteries are low. 

Some interviewees stated that ENDS users sometimes switched an internal battery with an external battery or upgraded from a smaller mod to a larger mod. Internal batteries often have lower wattage outputs and heat coils more slowly than external batteries. Bigger mods hold a larger number of batteries and are compatible with stronger batteries. Thus, users who wanted to produce larger clouds and experience stronger throat hits would change their batteries and mods to increase the devices’ wattage outputs. Caleb, a vape shop worker, said, “A lot of people that come in here and upgrade from an internal battery to an external battery, just for the bigger cloud, the harder hit… Once you don’t get the wattage that you like, you grab the bigger mod, you have ‘Big Beast’ now, you put batteries in, you’re going to get that huge cloud that you were trying to look for”.

Another common practice of battery modification is to install a battery in a device that was not designed for that battery. Participants expressed that sometimes they preferred a lower wattage output, below the designed wattage ranges of their devices. In those cases, they needed to install lower power batteries; yet, the battery sizes usually did not fit the devices, requiring them to put in “battery adapters”. Anthony described, “Pretty much that mod specifically is made to where it takes 2700 batteries. If I wanted to put an 18650 battery in there which is less power, I would have to do a build for that to pretty much feed that. And at the same time, 18650 wouldn’t fit into this bigger mod since it was made for 2700s. They have little adapters and little smaller pieces you would be able to say, ‘Okay, I want to put my battery in a sleeve and put it in my mod’, then it would work”.

#### 3.1.4. Chipsets

About half of the participants mentioned modifications to chipsets. Similar to those in computer systems, chipsets in ENDS devices include a set of electronic components that automate the process of charging and modifying the amount of power that the devices provide. Interviewees explained that chipsets are usually considered “higher-end” and provide an enhanced experience by allowing for automatic preheating, temperature control, and wattage control. The most commonly discussed modification was to change chipsets in order to alter the heating functions of the devices. Jacob mentioned that he replaced a chipset that offered only one wattage output with a new chipset that automatically increased wattage outputs as the device heated up. Adding manufactured chipsets to their own devices was also mentioned, but participants underlined that those modifications were often performed on devices not intended to be modified. Josh said, “I don’t think some manufacturers intend on having their devices altered, whether it’s dismantling the device and then people put in different chipsets. But there are a lot of people who actually try to build their own device. They take companies-like (manufactured) chipsets and they put it into a piece of wood, and they carve it out and build their own devices”.

#### 3.1.5. E-Liquids

More than half of the participants mentioned modifications to e-liquids. Three practices were commonly discussed, including refilling closed pods that are not meant by the manufacturer to be refilled, changing e-liquids with different flavors or nicotine levels, and producing their own by mixing different e-liquids or other substances. First, many users bought pre-made e-liquids to refill their closed pods. Reasons often mentioned were to lower the cost and increase control over the products they use. Participants also suggested that certain closed pods were easy to open, which encouraged them to refill the pods. Kayla said, “I can just use my fingertips and open up the device very easily. It’s very easy to do. And with JUUL, I usually use a tool to open this bottom part and add the juice. I’m mostly doing it so I can have better control on what product I use, and I can save the money. It’s a lot cheaper to buy a bottle of juice than buy separate pods”.

Another popular practice was to change different types of e-liquids. Participants mentioned that there are a lot of e-liquid options, which contain different flavors and various nicotine levels. Users often chose different flavors based on their personal tastes. Certain flavors that were perceived as better for ENDS devices and users’ health were also likely to be filled. Emily said, “We have some of the popular flavors that a majority of people will like, especially some of the juices that are less harsh on coils. And they’re made with all-natural flavorings. People tend to like that aspect of it, because they know they’re not getting a random chemical that tastes like this. It’s the actual fruit”. ENDS consumers also switch between different types of e-liquids so that they can control the amount of nicotine they vape and the throat hits. One product frequently mentioned was salt nicotine e-liquid. Compared to traditional e-liquids, salt nicotine e-liquids allow users to vape higher nicotine strengths while experiencing a less harsh throat hit [13]. Due to its high nicotine content, it also mimics the sensation of smoking a combustible cigarette. Thus, many participants mentioned changing to a salt nicotine product.

Creating one’s own e-liquids was also commonly mentioned. Some users mixed different manufactured products to create the unique e-liquids they liked. Noah, for instance, mentioned that he mixed a watermelon-flavored e-liquid with a salt nicotine product, so that he can enjoy the high nicotine and the fruit flavor. In addition, some users added other substances in their e-liquids such as cannabidiol (CBD) and THC or marijuana. Participants mentioned that although there were no devices specifically designed for vaping CBD or THC, users had used “cartridge devices” or “dab pens” to vape those substances. The devices resemble a small pen with a cartridge at the top and a battery at the bottom. Users can take the top off and refill the cartridge with any type of e-liquids or install another cartridge that contains CBD or THC in it. Charlotte said, “I have CBD products that you would put into any of these devices, but specifically for a device made for that, no”. Jack mentioned, “There are some batteries which are cartridge devices, where people can put liquids which have THC in it, or, as you probably know, dab pens. These devices were not manufactured for that use, but a lot of people have used it in that sort of aspect”.

According to the interviewees, the major reason that they add CBD to their e-liquid is for medical purposes as CBD helps reduce their pain from physical conditions. Beth stated, “I use CBD because I have rheumatoid arthritis. So instead of taking all the medicine, I use the CBD oils every day to control my flare-ups with rheumatoid”. Other participants mentioned that they vaped to quit smoking, and CBD helped them overcome negative reactions to medical treatment. Charlotte said, “I got diagnosed with breast cancer, and I was a smoker. I knew I was going to have withdraws (withdrawals) from the nicotine. So I came and talked to the guys here, who are the guys that manage the store, and started vaping, and went to my chemo and started adding CBD on a regular basis to help me with the nausea, the vomiting, the being able to eat”.

### 3.2. Trend in Users’ Modifications to ENDS over Time

A change in the popularity of ENDS modifications emerged from the interviews. Specifically, participants underlined that ENDS modifications, especially modifications to coils and batteries, were more widespread a couple years ago, but today, a smaller proportion of users engaged in modifications. Jacob said, “Two or three years ago, even four years ago, the only option you had to run high wattage was to build your own course. That has changed. Not many people do any modifications. There is a small hobby group, but I mean small hobby group compared to the average person”. Alex also stated, “There’s still a market. There’re still people going to cloud competitions … but it’s very small niche now compared to where it was two years ago”.

Participants attributed the decline in ENDS modifications to the evolution of manufactured devices. As Emily mentioned, “The products that have the pre-made coils, which is the main thing you would modify in a device like that, have gotten so much better, that there’s really not a need to do that”. Similarly, Jacob said, “You don’t get a lot of edge out of building your own coils like you used to. Coils were so bad previously… now if it’s not a hobby, you’re not going to do it”. Moreover, Charlotte applauded her current device, “It’s rubberized. It’s waterproof. I mean they come up in style and you’re not going to break it”. Jack liked the small size of his JUUL, “So you can take one of those little devices and put it in your pocket and carry it easier… So the smaller the device, the easier it is to conceal and the easier it is to carry”.

Those examples demonstrated that instead of merely updating their products, the industry has actively developed new products and modified their designs so that they can fit and foster users’ various needs. Emily highlighted, “And they really revolutionized a lot of products on the market. And their products have always been really great products. Stand up to the test of time. They’re durable. … If there’s a need in the vaping market, they’ll try to find a product, or make a product to fit that need”. Ultimately, Jacob felt that “Yeah, you could ride a horse everywhere, but if you’ve got a car, you’re probably just going to drive”. In other words, while users’ ENDS modifications were still prevalent, industry-led modifications have increasingly dominated the market, making users’ modifications to coils and batteries less necessary.

## 4. Discussion

Given the growing concerns about health effects of ENDS [6,7,8] and emerging evidence that the modifications to ENDS may result in even more health risks [9], our study aimed to begin filling the gap about how and why users modify ENDS. In our in-depth interviews with ENDS enthusiasts, we found that ENDS modifications primarily relate to the operational characteristics of the devices and e-liquids used in the devices. Users want to produce bigger clouds or do cloud tricks. They also desire to control the tastes of e-liquids, nicotine delivery levels, and throat hits. Thus, the most common modifications discussed by these participants were related to coils, batteries, and e-liquids.

A novel finding is that the prevalence of ENDS modifications might have peaked a couple of years ago. Participants mentioned that reliable, durable, and powerful devices had been largely absent from the market and to achieve higher power, a specific coil build, or a desired cloud density, users had to modify or build their own devices. However, over the last several years, manufacturers have caught up to the needs of the market, and instead of improving a device or building one from scratch, users can now buy premade products that meet their specific needs. 

In addition, JUUL and other pod-based ENDS have gained popularity in recent years. While users previously had to resort to artisanal ways to increase the nicotine delivery, such as by increasing the power of their devices or modifying the coil, salt nicotine pods afforded quick, convenient, and strong nicotine delivery, removing the need for user modifications. Notably, while average users may be less likely to alter their devices, those who do modify ENDS tend to be hobbyists and their modifications could be more extreme, resulting in higher health risks and more potential dangers.

The findings also revealed users’ modifications to e-liquids, which directly affect the tastes, flavors, and nicotine levels that users experience. Despite the view that fewer average consumers modify coils and batteries, many participants pointed out that modifications of e-liquids, such as mixing different e-liquids and adding substances to the e-liquids, is widespread among users. Notably, although participants attributed the use of CBD to medical reasons, they rarely explicitly explained the use of THC but suggested that adding THC was mostly for recreational purposes. In addition, interviewees also acknowledged that there were no devices specifically designed and manufactured for vaping CBD and THC, raising the safety concerns of using those products.

### 4.1. Implications for Tobacco Regulation

As of September 2019, New York and Michigan have banned the sale of flavored ENDS. On 2 January 2020, the FDA issued final guidance that indicated their highest enforcement priority was against any “flavored, cartridge-based ENDS product (other than tobacco- or menthol-flavored ENDS product)”. This policy indicates that they intend to “clear the shelves” of non-tobacco flavored ENDS, but will consider, as applications are made to FDA, whether these products are appropriate for the protection of public health and thus, meet the standard for marketing as new tobacco products.

The results from our study have indicated that this group of ENDS enthusiasts has made a wide range of modifications to ENDS in order to achieve desired nicotine and flavor delivery and enable the generation of noticeable aerosol clouds. However, because manufacturers are providing products that meet the desired characteristics, participants indicated that the interest in consumer modification of products has waned compared to only a few years ago. FDA enforcement actions to remove manufactured ENDS with certain characteristics from the market could reinvigorate the enthusiasm for consumer modification. The FDA needs to carefully consider how to minimize the unintended consequences of its regulatory actions that shrink product variety or remove products with specific characteristics. Otherwise, consumer modifications could increase, and additional outbreaks may become increasingly more common as consumer modification resurges.

### 4.2. Limitations

The qualitative nature of the study limits the generalizability of the findings. Future quantitative research should examine the health and social impacts of ENDS modifications at the population level. We only examined the views of how and why ENDS users modify their devices among a limited group of ENDS enthusiasts. Other aspects of ENDS modifications, such as information sources, users’ perceptions of ENDS modifications, and whether those modifications actually met the needs of ENDS enthusiasts, remain underexplored and should be addressed by future research. Only three participants were young adults (18–24 years). As ENDS use may damage teens’ and young adults’ brain development and result in other health harms [14], more studies are needed to examine how ENDS devices’ modifiable features and actual modifications contribute to the initiation of ENDS use among youth.

## 5. Conclusions

Among this group of enthusiasts, users’ modifications to ENDS focus on operational characteristics of the devices and e-liquids. They indicated that because the industry has constantly updated and developed new products to satisfy consumers’ needs, a smaller number of users seem to be altering their coils, batteries, and other operational features of ENDS in recent years. Yet, those who continue to modify their devices tend to be hobbyists, who perform more risky modifications, and users continue to misuse or abuse e-liquids. FDA should be aware of users’ modifications to ENDS as they consider related regulatory decisions and assess the resulting population health impact.

## Figures and Tables

**Table 1:** Demographic information, tobacco use, and electronic nicotine delivery systems (ENDS) modification practices of each participant.

Pseudonym	Sex	Age	Race	Education	Annual Income	Vaping Initiation	Tobacco Products Ever Used	ENDS Modifications Ever Done or Mentioned in the Interview
Alex	Male	25–44	Other	Some college	I prefer not to say.	2014	Cigarettes, Cigars, ENDS, Hookah, Others	Coils, Cotton wicks, E-liquid, Power control
Anthony	Male	18–24	Black	Some college	$25,000–$34,999	16 years old	ENDS	Coils, Cotton wicks, E-liquid, Power control
Beth	Female	25–44	Black	Bachelor’s degree	$75,000–$99,999	Not reported	ENDS, Hookah, Others	Power control
Caleb	Male	18–24	White	High school graduate	$100,000–$149,999	2014	ENDS, Hookah	Coils, Cotton wicks, E-liquid, Power control
Charlotte	Female	45–64	White	Bachelor’s degree	$35,000–$49,999	2016	Cigarettes, ENDS, Hookah, Others	Coils, Cotton wicks, E-liquid, Power control
Daniel	Male	25–44	Black	Some college	$35,000–$49,999	2012	Cigarettes, ENDS, Hookah	E-liquid, Power control
Emily	Female	25–44	White	Bachelor’s degree	$25,000–$34,999	2014	Cigarettes, ENDS, Hookah	Coils, Cotton wicks, E-liquid, Power control
George	Male	18–24	Asian	High school graduate	$35,000–$49,999	Not reported	ENDS, Hookah	Coils, E-liquid, Power control
Jack	Male	45–64	Black	Some college	$50,000–$74,999	“A long time ago”	Cigarettes, ENDS, Hookah, Pipes, Rolled your own tobacco products, Others	Coils, Cotton wicks, E-liquid, Power control
Jacob	Male	25–44	White	Bachelor’s degree	$50,000–$74,999	2011	Cigarette, ENDS; Hookah, Pipes, Rolled your own tobacco products, Others	Coils, Power control
Josh	Male	18–24	Asian	High school graduate	$35,000–$49,999	13 years old	Cigarettes, ENDS, Hookah	Coils, Cotton wicks, Power control
Kayla	Female	25–44	Black	Bachelor’s degree	$50,000–$74,999	2018	Cigarettes, ENDS Hookah	Coils, E-liquid, Power control
Noah	Male	25–44	Black	Graduate degree	$50,000–$74,999	2018	ENDS, Hookah, Others	Coils, Cotton wicks

## References

[1] Walley S.C., Wilson K.M., Winickoff J.P., Groner J. (2019). A public health crisis: Electronic cigarettes, vape, and JUUL. Pediatrics.

[2] Mirbolouk M., Charkhchi P., Kianoush S., Uddin S.M.I., Orimoloye O.A., Jaber R., Bhatnagar A., Benjamin E.J., Hall M.E., DeFilippis A.P. (2018). Prevalence and distribution of e-cigarette use among U.S. adults: Behavioral risk factor surveillance system, 2016. Ann. Intern. Med..

[3] Outbreak of Lung Injury Associated with E-Cigarette Use, or Vaping. https://www.cdc.gov/tobacco/basic_information/e-cigarettes/severe-lung-disease.html.

[4] Blount B.C., Karwowski M.P., Morel-Espinosa M., Rees J., Sosnoff C., Cowan E., Gardner M., Wang L., Valentin-Blasini L., Silva L. (2019). Evaluation of bronchoalveolar lavage fluid from patients in an outbreak of e-cigarette, or vaping, product use–associated lung injury—10 states, August–October 2019. MMWR Morb. Mortal. Wkly. Rep..

[5] Weaver S.R., Huang J., Pechacek T.F., Heath J.W., Ashley D.L., Eriksen M.P. (2018). Are electronic nicotine delivery systems helping cigarette smokers quit? Evidence from a prospective cohort study of U.S. adult smokers, 2015–2016. PLoS ONE.

[6] National Academies of Sciences, Engineering, and Medicine (2018). Public Health Consequences of E-Cigarettes.

[7] Goniewicz M.L., Smith D.M., Edwards K.C., Blount B.C., Caldwell K.L., Feng J., Wang L., Christensen C., Ambrose B., Borek N. (2018). Comparison of nicotine and toxicant exposure in users of electronic cigarettes and combustible cigarettes. JAMA Netw. Open.

[8] McConnell R., Barrington-Trimis J.L., Wang K., Urman R., Hong H., Unger J., Samet J., Leventhal A., Berhane K. (2016). Electronic cigarette use and respiratory symptoms in adolescents. Am. J. Respir. Crit. Care Med..

[9] Brown C.J., Cheng J.M. (2014). Electronic cigarettes: Product characterisation and design considerations. Tob. Control.

[10] Talih S., Balhas Z., Salman R., Karaoghlanian N., Shihadeh A. (2016). “Direct dripping”: A high-temperature, high-formaldehyde emission electronic cigarette use method. Nicotine Tob. Res..

[11] Coenen M., Stamm T.A., Stucki G., Cieza A. (2012). Individual interviews and focus groups in patients with rheumatoid arthritis: A comparison of two qualitative methods. Qual. Life Res..

[12] Braun V., Clarke V. (2006). Using thematic analysis in psychology. Qual. Res. Psychol..

[13] Jackler R.K., Ramamurthi D. (2019). Nicotine arms race: JUUL and the high-nicotine product market. Tob. Control.

[14] U.S. Department of Health and Human Services (2016). E.-Cigarette Use Among Youth and Young Adults: A Report of the Surgeon General.

